# Protecting and promoting the rights of the ‘reserve army of labour’: a policy analysis of structural determinants of migrant worker health in Pakistan and Qatar

**DOI:** 10.1093/heapol/czad029

**Published:** 2023-05-10

**Authors:** Sarah Hawkes, Mireille Evagora-Campbell, Aysha Zahidie, Fauziah Rabbani, Kent Buse

**Affiliations:** Institute for Global Health, University College London, 30 Guilford Street, London WC1N 7HT, United Kingdom; Institute for Global Health, University College London, 30 Guilford Street, London WC1N 7HT, United Kingdom; Department of Community Health Sciences, Aga Khan University, PO Box 3500, Stadium Road, Karachi 74800, Pakistan; Department of Community Health Sciences & Associate Vice Provost Research & Graduate Studies, Aga Khan University, PO Box 3500, Stadium Road, Karachi 74800, Pakistan; Healthier Societies Program, The George Institute for Global Health, Imperial College London, 84 Wood Lane, London W12 0BZ, United Kingdom

**Keywords:** Labour migrant health, social determinants, structural determinants, HIV, COVID-19, global guidance, policy content cubes

## Abstract

Labour migrants who travel overseas for employment can face deep health inequities driven in large part by upstream social and structural determinants of health. We sought to study the ‘labour migrant health ecosystem’ between one sending country (Pakistan) and one host country (Qatar), with a focus on how the ecosystem realizes the rights of labour migrants when addressing the social and structural determinants (e.g. housing, employment law, etc.) of health. Study objectives were to (1) undertake an in-depth review of policies addressing the structural and social determinants of the health of labour migrants in both Pakistan and Qatar, analysing the extent to which these policies align with global guidance, are equity-focused and have clear accountability mechanisms in place, and (2) explore national stakeholder perspectives on priority setting for labour migrant health. We used a mixed methods approach, combining policy content analysis and interviews with stakeholders in both countries. We found a wide range of guidance from the multilateral system on addressing structural determinants of the health of labour migrants. However, policy responses in Pakistan and Qatar contained a limited number of these recommended interventions and had low implementation potential and minimal reference to gender, equity and rights. Key national stakeholders had few political incentives to act and lacked inter-country coordination mechanisms required for an effective and cohesive response to labour migrant health issues. Effectively addressing such determinants to achieve health equity for labour migrants will depend on a shift in governments’ attitudes towards migrants—from a reserve army of transient, replaceable economic resources to rights-holding members of society deserving of equality, dignity and respect.

Key messages

- The implementation of effective and equitably structural interventions to improve the health of labour migrants represents a double whammy of neglect: a neglected level of intervention and a neglected population. This is even though labour migrants account for the largest proportion of all migrants globally.
- Our review finds a relatively large number of global recommendations for structural interventions to improve the health of labour migrants. However, policy responses in one ‘sending’ country (Pakistan) and one ‘host’ country (Qatar) are found to contain a limited number of these global recommendations, and national policies have low implementation potential and minimal reference to consideration of equity and rights.
- Labour migrants are primarily viewed as a replaceable economic resource instead of rights-holding members of society, which diminishes the priority given to migrant health policies in both countries.
- National decision makers lack political incentives to act on migrant health and have not established mechanisms for inter-sectoral or inter-state action. Whether national policymakers address these policy gaps will depend to some extent on effective mobilization of migrant workers but will also necessitate systems of independent monitoring and accountability from the global community.

## Introduction

Labour migration is big business. Here we report on findings from a multi-methods study of what we are calling the ‘labour migrant health ecosystem’ between one sending country (Pakistan) and one host country in the GCC—Qatar. We focus on the structural determinants of the health and well-being of labour migrants—which includes, e.g. determinants in the fields of labour rights, housing, etc. We examine the extent to which policies (global, national and bilateral) addressing structural determinants seek to protect and promote policies and interventions to realize positive health outcomes for labour migrants and seek to explain our policy analysis findings through in-depth interviews with key stakeholders in both countries.

In 2020, over 200 000 Pakistanis sought employment overseas (Government of Pakistan, 2020), a number presumably affected by COVID-19 and lower than the average of more than 560 000 leaving for overseas work annually during the period 2008–2015 (GIZ (Deutsche Gesellschaft für Internationale Zusammenarbeit) and International Labour Organization, 2016). Overseas Pakistanis sent US$23.13 billion in remittances (Government of Pakistan, 2020), accounting for almost 9% of Pakistan’s GDP (World Bank, 2022). Over 96% of Pakistani international labour migrants workers, defined as ‘migrants of working age, who during a specified reference period, were in the labour force of the country of their usual residence, either in employment or in unemployment’ (ILO, 2021a), have found employment in the six Gulf Cooperation Council (GCC; Kingdom of Bahrain, Kingdom of Saudi Arabia, State of Kuwait, State of Qatar, Sultanate of Oman and United Arab Emirates) countries during the past 50 years (ILO, 2020). The vast majority (over 98%) of Pakistani labour migrant workers in Qatar are men (GIZ (Deutsche Gesellschaft für Internationale Zusammenarbeit) and International Labour Organization, 2016).

Qatar, a GCC country, has undergone rapid economic development in the past several decades driven by wealth from gas and oil exports (IOM, 2019; World Bank, 2020), and 95% of its labour market is made up of migrants (Ewers *et al.*, 2020). The large-scale infrastructure projects launched in preparation for the Federation Internationale de Football Association (FIFA) World Cup 2022 (Georgetown University & Qatar Foundation, 2019) and the development ambitions set out in the Qatar National Vision 2030 (Shah, 2017) have generated a rise in the population of migrant workers, especially in construction, manufacturing and domestic services (IOM, 2019). However, as Babar and Vora point out, the reliance of the Arabian Peninsula on ‘externally sourced labour to meet the needs of various sectors and industries’ has a long history entwined with the even longer histories of colonialism and slavery (Babar and Vora, 2022). For example, both the pearl fishing and gas/oil extraction industries that contributed to the economies of the Gulf region (including Qatar) created ‘demand for a cheap, biddable workforce’—a workforce that, historically, has been characterized by both national and racial categories (Vora and Le Renard, 2021; Babar and Vora, 2022).

Contemporary labour migrants are sometimes referred to as ‘blue collar’, ‘low-skilled’ or ‘semi-skilled’ and may be distinguished in Qatari policies from ‘white collar’, ‘high-skilled’ or ‘professional’ migrants and granted different legal status and rights on the basis of their salary (Qatar Ministry of Development Planning and Statistics, 2018). They are predominantly male (83% of Qatar’s migrant population) (ILO, 2016a) and young (median age of 33.4 years) (De Bel-Air, 2018)—constituting the so-called ‘single male labourers’ (Migration Data Portal, 2021).

Notwithstanding the ‘healthy migrant effect’—which sees arriving migrants generally being relatively young, fit and in good health (Chaabna *et al.*, 2017; Amnesty International, 2021)—there are health inequities between nationals and the migrant population in Qatar. Nearly 7000 deaths of migrants from five labour-sending countries were documented between 2010 and 2020 (Pattisson *et al.*, 2021). Research on the prevalence of depressive symptoms among migrants and non-migrants in Qatar found a significant increase in odds ratios for depression among labour migrants compared to non-migrants (and also compared to white-collar migrants), and this risk was associated with having a problem with the current employer (Khaled and Gray, 2019). In the early stages of the COVID-19 pandemic, a study of antibody seroprevalence among the urban population presenting for health care in Qatar found higher rates among Pakistanis and lower rates among Qataris compared to other nationalities (Alishaq *et al.*, 2021). Another, smaller study in this early pandemic stage found that seroprevalence among transport workers, cleaning workers, technical and construction workers and security workers was higher than that of ‘professional workers’ (Al-Thani *et al.*, 2021).

Health outcomes of labour migrants are influenced by structural and social determinants (Egli-Gany *et al.*, 2020; Evagora-Campbell *et al.*, 2022)—a wide-ranging set of conditions that encompass, inter alia, legal policies and commercial drivers. In Qatar, for example, a study by Ewers and colleagues on migrant workers’ well-being found that ‘contracts [were] the most important determinant of migrant worker welfare’, where contracts encompassed systems of recruitment and sponsorship systems as well as written documentation (often not in the worker’s native language) and lax enforcement. Employment conditions, including job-associated housing, can be associated with poor health outcomes, including through sleep-deprivation, dehydration resulting from heat exposure, unsanitary working conditions, stress and on-site injuries (Georgetown University & Qatar Foundation, 2019). Additionally, housing is often in shared communal spaces, and in 2020, the first documented community transmission of SARS-CoV-2 in Qatar was linked to a cluster among 300 migrant ‘craft and manual’ workers living in this type of high-density housing (Abu-Raddad *et al.*, 2021). Moreover, these migrants often live in financially precarious conditions: over 60% of migrants earn less than 1500 QR (USD$411) per month from their primary job, and it has been estimated that 90% are in debt from the financial costs of migration (Ewers *et al.*, 2020), with widespread salary abuses also reported (Human Rights Watch, 2020).

To date, there has been limited in-depth analysis of how sending and host countries (in this case, Pakistan and Qatar, respectively) within the labour migrant ecosystem are addressing the structural determinants of labour migrant health, either individually or collaboratively, raising questions about the onus of responsibility and mechanisms of accountability. Our study of the labour migrant health ecosystem set out to investigate the extent to which policies and actors within the ecosystem serve to promote and protect ‘healthy conditions’ for labour migrant health and well-being. In the case of the host country, the policies impact directly on the structural drivers of health and well-being. In the case of the sending country, however, policies are more generally those that impact on the ability of the country itself to influence whether their citizens’ rights in a host country are met and health-promoting environments are supported and sustained.

Pakistan and Qatar both voted in favour of a 2018 resolution to endorse The Global Compact for Safe, Orderly and Regular Migration, a non-binding agreement between UN Member States (United Nations, 2018; ILO, 2020). However, there are several legally binding commitments critical to migrant workers, which neither country has ratified, including the OHCHR Convention on the Protection of the Rights of All Migrant Workers and Members of Their Families (OHCHR, 2021), the ILO Migration for Employment Convention (ILO, 2021b) and the ILO Migrant Workers Convention (ILO, 2021c).

Pakistan has no formal labour emigration policy (ILO, 2020), and the existing legal framework for overseas labour recruitment, the 1979 Emigration Ordinance (purely a legal instrument, rather than a policy), fails to address some key issues, including skills training, the rights of workers overseas and research and data collection on the labour market. It also relies in a large part on private sector overseas employment promoters to facilitate overseas employment, including managing recruitment, departure and return for the majority of migrant workers (ILO, 2020).

In Qatar, commitments have been made towards ensuring necessary structures are in place to support the health of migrants, including Qatar’s second National Health Strategy (2018–2022), which identified ‘healthy and safe employees’ as a priority population (Georgetown University & Qatar Foundation, 2019). Significant attention for migrant health came in response to widespread criticism from the United Nations, human rights organizations, the media and governments [including that of Pakistan (Shabbir, 2020)] of Qatar’s treatment of migrant labourers following the award of the World Cup in 2010 (Georgetown University & Qatar Foundation, 2019).

Qatar’s use of the ‘*kafala*’ system of sponsorship, which had previously delegated a great deal of responsibility for migrant workers to private sector actors and granted legal power to employers to control workers’ employment decisions, travel and residency (ter Haar, 2018; Amnesty International, 2020), had raised considerable human rights concerns for migrant workers (Garrett, 2020). Under *kafala*, e.g. a survey of more than a thousand low-income workers in Qatar found widespread abuses including over half of respondents lacking a government health card and a fifth whose salary had been withheld (Gardner *et al.*, 2013). In 2015, the International Labour Office reviewed a complaint that was highly critical of the Government of Qatar: ‘From the moment migrant workers begin the process of seeking work in Qatar, they are drawn into a highly exploitative system that facilitates the exaction of forced labour by their employers … The Government of Qatar fails to maintain a legal framework sufficient to protect the rights of migrant workers consistent with international law and to enforce the legal protections that currently do exist’ (ILO, 2016c).

This system has been undergoing reforms since 2017 (Abraham, 2021; ILO, 2021d), and in March 2021, the No-Objection Certificate that workers were required to obtain from an employer in order to change jobs (Garrett, 2020; IOM, 2020) was abolished. Additional reforms include scrapping the ‘exit permit’ for the majority of migrant workers that required their employer’s permission to leave the country (Amnesty International, 2018) and introduction of a minimum wage (Qatar Government Communications Office, n.d.). Migrant workers (including labour migrants) were also protected through the introduction of the wage protection systems in 2015 (Jureidini, 2017b).

Progress has recently been made with respect to the health and well-being of labour migrants in Qatar, including through labour and employment reforms. Along with the removal of *kafala*, the Qatari Government has also sought to address inequities in health-care access, which had seen 40% of labour migrants with no health insurance card, compared to 6% of ‘white collar’ migrants and 2% of Qataris (Liu *et al.*, 2020). A law passed by the Shura Council in 2021 mandates employers to provide all employees, including non-Qatari nationals, with basic health insurance coverage (Qatar MOPH, 2021). It has been argued that the COVID-19 pandemic itself may have provided an impetus to policy reform as it ‘necessitated an unprecedented expansion of medical care and treatment to migrants’ (Ewers *et al.*, 2023).

However, despite these positive legal and policy reforms, working conditions of migrants frequently remain poor, employment rights abuses are reported to persist and stringent measures limiting migrants’ ability to change employers led Amnesty International to argue that 2021 saw ‘an actual erosion of newly protected migrant workers’ rights’ (Amnesty International, 2021).

## Objectives

As a partnership of researchers based in Pakistan and the UK, and in collaboration with colleagues in Qatar, our research has focused on understanding the ‘labour migrant health ecosystem’ of labour workers moving from Pakistan to Qatar. The objectives of our study were to (1) undertake an in-depth review of policies addressing the structural and social determinants of the health of labour migrants in both Pakistan and Qatar, analysing the extent to which these policies align with global guidance, are equity-focused and have clear accountability mechanisms in place, and (2) explore national stakeholder perspectives on priority setting for labour migrant health in order to consider the opportunities for, and prospects of, enhancing individual countries’ and collective responsibilities to protect the health of labour migrants.

## Methods

We used a mixed methods approach, combining policy content analysis and interviews with stakeholders in both countries. We reviewed policies relevant to labour migrant health in general and additionally included two tracer health issues—HIV/sexually transmitted infections (STIs) and COVID-19 infections. We focused on communicable diseases as these are more likely to be prevalent (e.g. compared to non-communicable diseases) in the age group of labour migrants (Chaabna *et al.*, 2017; Amnesty International, 2021). In addition, by looking at HIV/STIs and COVID-19, we were able to examine policy responses to both a protracted health concern and an emergency situation.

### Policy content analysis

We used a ‘policy cube’ approach, previously developed by some of the authors of this paper, (Buse *et al.*, 2020) to analyse policy content. Analysis covers three axes (i) ‘comprehensiveness’, i.e. the extent to which national policies reflect global evidence-informed recommendations, (ii) ‘values’, i.e. the inclusion of human rights and equity, including gender, in the policy and (iii) ‘implementation potential’, i.e. the presence of a budget line item, presence of systems of accountability and level of policy authority (stringency).

To construct a baseline for ‘comprehensiveness’, we reviewed ‘global guidance’ documents from multilateral organizations, as well as international agreements, to identify a list of recommendations agreed at the global level that address social and structural drivers of migrant labour health—see Table 1. Global guidance documents were identified by searching the online databases of UN entities (OHCHR, ILO, IOM and WHO), multilateral institutions (e.g. World Bank), UN conventions and other UN resources (reports of UN Special Rapporteurs) for guidance relating to health, migration or labour rights. Global documents were reviewed to identify recommendations addressing social and structural drivers of migrant labour health targeted at national governments.

**Table 1.** Policy recommendations from global level and their presence in policy documents of Pakistan and Qatar

	Applies to host/sending/both countries	Present in national policies in Pakistan (sending country)	Present in national policies in Qatar (host country)
*Part 1: Global guidance for health of labour migrants: Source documents*			
1. Cooperation between governments, workers’ organizations and employers’ organizations on managed labour migration and health programmes.^e^^,^ ^f^^,^ ^j^^,^ ^k^^,^ ^m^^,^ ^p^^,^ ^o^ ^t^^,^ ^u^	Both		Yes
2. Health data disaggregated by migratory status.^k^^,^^t^^,^^l^^l^			Yes
3. National labour migration and public health laws and policies that are aligned with international human rights standards.^a^^,^^e^^,^^f^^,^^h^^,^^j^^,^^k^^,^^l^^,^^m^^,^^t^^,^^u^^,^^r^^,^^aa^		Yes	Yes
4. Consultation of employers’ and workers’ organizations, civil society and migrant associations in labour migration policy development.^b^^,^^f^^,^^h^^,^^k^^,^^r^		Yes	Yes
5. Enforcement of national laws and regulations in line with international labour standards for nationals and non-nationals.^k^^,^^l^^,^^u^^,^^cc^			Yes
6. Regulation of recruitment and placement services to protect migrant workers and prevention of discrimination in recruitment processes.^b^^,^^c^^,^^h^^,^^i^^,^^k^^,^^n^^,^^o^^,^^p^^,^^t^^,^^u^^,^^cc^			
7. Financial inclusion of migrants including through minimum wage scales and restricting recruitment, remittance and travel fees.^b^^,^^c^^,^^t^^,^^u^^,^^p^^,^^cc^		Yes	Yes
8. Promotion of regular and equitable migration including by expanding and diversifying legal migration channels and combating smuggling, trafficking and forced labour.^b^^,^^f^^,^^h^^,^^k^^,^^p^^,^^q^^,^^s^^,^^t^			Yes
9. Access to grievance mechanisms for migrants to address labour rights abuses.^o^^,^^u^^,^^x^^,^^y^^,^^aa^^,^^bb^			
10. Information for migrants on their rights and legal entitlements relating to recruitment and employment conditions.^e^^,^^f^^,^^o^^,^^p^^,^^t^^,^^u^^,^^y^			Yes
11. Measures to address gender disparities in migrant worker movements and gender barriers affecting access to information and health care.^j^			
12. Accessible public health information targeted at migrants including workplace programs^m^^,^^r^		Yes	Yes
13. Measures to protect migrant workers and their families during outward and return journeys and support during repatriation and integration.^b^^,^^e^^,^^f^^,^^k^^,^^t^		Yes	
14. Labour and public health laws and policies that do not discriminate based on infectious disease status including ending mandatory testing practices and restrictions on entry, stay and residence.^l^^,^^m^^,^^r^		Yes	
15. Guarantee of right of association and freedom for all lawful trade union activities.^e^^,^^h^^,^^u^		Yes	
16. Measures to promote inclusion and combat racism, xenophobia and intolerance against migrants.^k^^,^^y^	Host	N/A	Yes
17. Measures to address economic, administrative, linguistic and cultural barriers to migrant workers’ access to health-care services.^c^^,^^e^^,^^j^^,^^l^^,^^r^^,^^s^^,^^t^		N/A	Yes
18. Maintenance of occupational health and safety standards and decent working conditions including through labour inspections and information for employers on good employment practices.^o^^,^^s^^,^^t^^,^^bb^^,^^ee^		N/A	Yes
19. Safe, sanitary living conditions with adequate infection prevention and control measures.^b^^,^^f^^,^^w^^,^^bb^^,^^dd^^,^^ee^		N/A	Yes
20. Access to social protection schemes for migrant workers and their families.^b^^,^^d^^,^^e^^,^^f^^,^^g^^,^^h^^,^^k^^,^^s^^,^^t^^,^^o^^aa^^,^^dd^^,^^kk^		N/A	Yes
*Part 2: Global guidance for addressing health of labour migrants during COVID-19 pandemic: Source documents*			
1. Limited emergency powers in line with international law.^bb^^,^^dd^^,^^ee^^,^^ff^^,^^gg^	Both		Yes
2. Administrative measures to support undocumented and stranded migrants and ensure safe movement within and between countries including temporary work permits.^v^^,^^ee^			Yes
3. Emergency financial support for migrants.^y^^,^^aa^^,^^ii^			
4. Support for remittance infrastructure during the crisis.^ee^^,^^j^^j^			
5. Financial support to businesses that employ migrants conditional on keeping migrants employed.^hh^	Host	N/A	
6. Release of migrants from immigration detention centres.^dd^^,^^ee^		N/A	
7. Firewalls between immigration enforcement and public health services and protect the privacy rights of migrants.^y^^,^^ee^		N/A	

Key for Table 2.

a ILO Social Security (Minimum Standards) Convention (No. 102), 1952.

b ILO Protection of Migrant Workers (Underdeveloped Countries) Recommendation (No. 100), 1955.

c ILO Plantations Conventions (No. 110), 1958.

d ILO Equality of Treatment (Social Security) Convention (No. 118), 1962.

e ILO Convention concerning Migration for Employment (Revised) (No. 97), 1949.

f UN International Convention on the Protection of the Rights of All Migrant Workers and Members of Their Families, 1990.

g ILO Maintenance of Social Security Rights Convention (No. 157), 1982.

h ILO Convention concerning Migrations in Abusive Conditions and the Promotion of Equality of Opportunity and Treatment of Migrant Workers (Supplementary Provisions), 1975 (No. 143).

i ILO Private Employment Agencies Convention (No. 181), 1997.

j ILO and UNAIDS Migrants’ Right to Health guidelines, 2001.

k ILO Multilateral Framework on Labour Migration, 2006.

l UNAIDS HIV and International Labour Migration Policy Brief, 2008.

m ILO Recommendation concerning HIV and AIDS and the World of Work, 2010.

n ILO Domestic Workers Convention, (No. 189), 2011.

o ILO Domestic Workers Recommendation (No. 201), 2011.

p Forced Labour (Supplementary Measures) Recommendation (No. 203), 2014.

q Protocol to the 1930 ILO Forced Labour Convention, 2014.

r UNAIDS GAP report 2014.

s ILO Promoting a Rights-based Approach to Migration, Health, and HIV and AIDS: A Framework for Action, 2016.

t UN Global Compact on Migration 2018.

u ILO General principles and operational guidelines for fair recruitment, 2019.

v FAO migrant workers and the COVID-19 pandemic, 2020.

w IASC, FRC, IOM, UNHCR and WHO, Interim Guidance Scaling-Up Covid-19 Outbreak Readiness and Response Operations in Humanitarian Situations, Including Camps and Camp-Like Settings, 2020.

x International Labour Conference 2020: Promoting fair migration, 2020.

y ILO ensuring fair recruitment during the COVID-19 pandemic, 2020.

z ILO protecting migrant workers during COVID-19 pandemic Recommendations, 2020.

aa IOM guidelines for labour recruiters on ethical recruitment, 2020.

bb IOM migrants and the COVID-19 pandemic: An initial analysis, 2020.

cc IOM Montreal Recommendations on Recruitment, 2020.

dd OHCHR COVID-19 and the Human Rights of Migrants: Guidance, 2020.

ee UN Committee on Migrant Workers and the UN Special Rapporteur on the Human Rights of Migrants Joint Guidance Note on the Impacts of the COVID-19 Pandemic on the Human Rights of Migrants, 2020.

ff UN Network on Migration COVID-19 & Immigration Detention: What Can Governments and Other Stakeholders Do?, 2020.

gg WHO Preparedness, prevention and control of coronavirus disease (COVID-19) for refugees and migrants in non-camp settings, 2020.

hh World Bank COVID-19 Crisis Through a Migration Lens Migration and Development Brief 34, 2020.

ii World Bank COVID-19 Crisis Through a Migration Lens Migration and Development Brief 35, 2020.

jj World Bank COVID-19 Crisis Through a Migration Lens Migration and Development Brief 36, 2020.

kk World Bank COVID-19 Crisis Through a Migration Lens Migration and Development Brief 37, 2020.

ll IOM The 2030 Agenda and Data Disaggregation, 2021.

Next, we reviewed national policies to evaluate the extent to which these contained recommendations aligned with the global guidance. We included three types of national policies from health and other ministries of the Pakistani and Qatari governments: (i) policies addressing migrant health generally (5 Pakistani policies and 15 Qatari policies); (ii) policies specific to HIV/STI outcomes among migrants (one Pakistani policy and five Qatari policies) and (iii) policies addressing COVID-19 outcomes and secondary impacts among migrants (seven Pakistani policies and eight Qatari policies). See Box 1 for details of the inclusion and exclusion criteria for national and global policies and Table 1 for evidence of global recommendations in national policies.

Box 1. Inclusion and exclusion criteria for policy documents

Global level documents

Inclusion criteria:

– Published by any entity of the UN system or other international, multilateral organization
– Addresses health, migration or labour rights
– Contains recommendations for addressing social or structural determinants of migrant health that are directed at national governments
– Available in English

Exclusion criteria:

– Not published by an entity of the UN system or other international, multilateral organization
– Does not address health, migration or labour rights
– Does not contain recommendations for addressing social or structural determinants of migrant health that are directed at national governments
– Not available in English

National level documents

Inclusion criteria:

– Published by the governments of Pakistan or Qatar^1^^,^^2^
– Relevant to the health of labour migrants (general health sample); HIV or STIs among labour migrants (HIV/STI sample); relevant to impact of COVID-19 on labour migrants^3^ (COVID-19 sample)
– Covers health or non-health sector interventions
– Available in English
– Applies at the national level

Exclusion criteria:

– Published by a non-government actor
– Not relevant to the health of labour migrants
– Not available in English
– Applies only at subnational level^4^

National policies were then reviewed for their attention to equity, gender and rights and for their implementation potential. All data were extracted from national policies into standardized Excel sheets by two reviewers working independently and reviewed for discrepancies by a third reviewer.

Results from analysing the national documents on criteria of comprehensiveness, equity and authority were then scored numerically in order to construct a policy cube for each country—see Table 2.

**Table 2.** Policy cube axis scoring methods

Axis	Component	Method for scoring component
1. Comprehensiveness	Comprehensiveness	Number of recommendations present in the policy sample: n/21 recommendations for Qatar non-COVID-19 policies n/15 recommendations for Pakistan non-COVID-19 policies n/29 for Qatar COVID-19 policies n/19 for Pakistan COVID-19 policies
2. Policy salience	Authority	1= national action plans, plans, strategies, guidelines, standards, action plans, directives, activities, conventions, contracts 2 = rules, regulations, by-laws 3 = constitutions, acts, laws, decrees
	Accountability	0 = accountability mechanism not mentioned 1 = national agency specified and assigned responsibility for reporting in the public domain 2 = national agency specified and assigned responsibility for reporting in the public domain, mechanism for independent monitoring on progress is described, remedial actions if implementation does not occur are outlined
	Budget	0 = no mention of financing 1= any mention of named financing body or resources allocated (such as budget line item)—for implementation of the policy
3. Equity, gender and rights	Equity	0 = no mention of equity or equality 1 = commitment to equity or equality, e.g. language around equal/universal access/opportunities 2 = commitment to equity or equality, e.g. language around equal/universal access/opportunities AND mention of specific groups that are considered vulnerable and will be targeted +1 if reference to equity relates to migrants
	Human rights	0 = no mention of human rights 1 = vague commitment to rights, legal entitlements, guarantee of just/humane treatment, human rights-based approach—but no reference to a specific human right or legal framework 2 = commitment to human rights in the context of a legal framework such as an international convention or treaty OR a specific human right that is recognized in international law +1 if reference to human rights relates to migrant
	Gender	Graded according to the *WHO Gender-Responsive Assessment Scale* 0 = gender blind 1= gender sensitive 2 = gender-specific 3 = gender transformative +1 if reference to gender relates to migrants

### Stakeholder interviews

The second part of our study was designed to assess stakeholder perceptions and positions on the policies within the labour migrant ecosystem. We conducted semi-structured interviews with national stakeholders to investigate two areas: (1) agenda-setting and policy-formulation factors contributing to the identified gaps in policy content and (2) opportunities to enhance the prospects that future policies address structural drivers of migrant labour health, including in ways that promote equity and gender responsiveness.

Stakeholder mapping was conducted in both Pakistan and Qatar in collaboration with key informants in both countries to identify stakeholders and potential interviewees. Informants were approached by email and interviewed either in-person or by zoom. In both countries, a number of stakeholders were approached but declined to be interviewed on this topic; in addition, in Qatar, in particular, we were limited in our ability to conduct interviews after the outbreak of the COVID-19 pandemic as key stakeholders were generally unavailable for interview.

We were not able to interview labour migrants themselves in either country as we lacked access and ethical permission for this. In total, we interviewed 13 stakeholders in Pakistan and 14 in Qatar, and these high-level interviewees represented a cross-section of sectors (health, non-health, public and private), government, civil society organizations, international organizations and media. Interviews in Qatar were conducted from October 2019 to February 2020 and covered issues relating to HIV/STIs and the health of labour migrants. Pakistan interviews were conducted from November 2019 to August 2021 and covered HIV/STIs, COVID-19 and the general health of labour migrants. Informed consent was obtained from all interviewees.

Interviews were recorded (with permission) and subsequently transcribed. In a small number of cases, only written notes were taken. Transcribed recordings and written notes were coded by hand using a predetermined framework of analysis based on Shiffman and Smith’s agenda-setting framework of four categories determining political priority among health issues: issue characteristics, ideas (i.e. the arguments, frames, narratives and critiques), actor power and political context (Shiffman and Smith, 2007).

We had hoped to conduct in-person round table policy dialogues with key policymakers and stakeholders in Pakistan and Qatar to discuss the analysis and identify opportunities for future policy change, but due to COVID-19 restrictions, this was not possible.

This research was approved by the ethics boards of the authors’ institutes and Weill Cornell Medical College Qatar (Qatar).

## Results

We identified 39 global guidance documents: 25 general health documents and 13 COVID-19 documents; 20 were technical and 18 were political documents. These included legally binding instruments such as conventions and treaties (*n* = 11); non-binding instruments such as recommendations and declarations (*n* = 6); technical guidelines (*n* = 10); reports (*n* = 2) and policy briefs (*n* = 9). We extracted 27 recommendations applicable at national level: 20 relating to general health and 7 recommendations specific to COVID-19. Eight of the 27 recommendations were targeted at receiving countries (e.g. ensuring occupational health and safety and decent working conditions including through labour inspections); 19 recommendations were targeted at both sending and receiving countries. None were targeted specifically at sending countries.

In Pakistan and Qatar, we identified 12 and 25 policies (respectively) relevant to the health of labour migrants—see Tables 3 (Pakistan) and 4 (Qatar). Policies were in the health sector (7 in Pakistan and 12 in Qatar) and sectors/ministries other than health (6 in Pakistan and 13 in Qatar).

**Table 3.** Government of Pakistan policies that contain elements to protect the health of labour migrants through attention to social and structural drivers (*N* = 12)

Policy name (and issuing authority)	Area of health covered in policy	Does it contain a global recommendation?	Does it contain elements of gender responsiveness (GR), equity (EQ) or human rights (HR)?	Level of policy authority level (AL, AM and AH), presence of accountability mechanism (AcW and AcS) or financing mechanism (F)^a^
Pakistan labour policy 2010 (Ministry of Labour)	General	Yes	GR, EQ, HR	AL
Emigration ordinance 1979 updated 2012 (Government of Pakistan)	General	No		AH
Emigration Rules 1979 updated 2019 (Government of Pakistan)	General	No		AM
Vision 2025 (Ministry of Planning, Development, and reform)	General	No	HR	AL
National Health Vision (2016–2025) (Ministry of National Health Services, Regulation and Coordination and Provincial Health Departments, Government of Pakistan)	General	No	EQ, HR	AL
Pakistan AIDS strategy III (2017–2022) (National AIDS Control Program)	STIs	Yes	GR, EQ, HR	AL, F
Advisory on Mitigation Strategies COVID-19 (Ministry of National Health Services)	COVID-19	No	HR	AL
Guidelines for Adult Vaccination Counters (AVCs) in Pakistan (Ministry of National Health Services)	COVID-19	No	EQ	AL
Social Distancing during COVID 19 Outbreak (Health Services)	COVID-19	No		AL
Pakistan Preparedness & Response Plan COVID-19 (Government of Pakistan)	COVID-19	No	GR, EQ, HR	AL, AcW
2019-nCoVirus Clinical Care & Prevention Gop Guidelines (Government of Pakistan)	COVID-19	No		AL
Testing Strategy Incorporating COVID-19 Antigen Detection Rapid Diagnostic Tests (Ag-RDT) (Government of Pakistan)	COVID-19	No		AL

a Policy authority level: AL = low policy authority (national action plans, plans, strategies, guidelines, standards, action plans, directives, activities, conventions, contracts, etc.); AM = medium policy authority (rules, regulations, policies, by-laws, conventions, etc.); AH = high policy authority (constitutions, acts, laws, decrees etc.).

Presence of accountability mechanism: AcW = weak accountability mechanism present (national agency specified and assigned responsibility for reporting in the public domain), AcS = strong accountability mechanism present (national agency specified and assigned responsibility for reporting in the public domain, mechanism for independent monitoring on progress is described, remedial actions if implementation does not occur are outlined).

Presence of financing mechanism: F = financing mechanism present (mention of named financing body or resources allocated, such as budget line item, for implementation of the policy).

**Table 4.** Government of Qatar policies that contain elements to protect the health of labour migrants through attention to social and structural drivers (*N* = 25)

Policy name (and issuing authority)	Area of health covered in policy	Does it contain a global recommendation?	Does it contain elements of gender responsiveness (GR), equity (EQ) or human rights (HR)?	Level of policy authority level (AL, AM and AH), presence of accountability mechanism (AcW and AcS) or financing mechanism (F)^a^
National Health Strategy 2018–2022: Our health, our future (Ministry of Public Health)	General	Yes	HR	AL, F
Occupational Health and Safety Strategy (Laboratories and Standardization Affairs and Qatar General Organization for Standards and Methodology)	General	Yes	HR	AL
Qatar Healthcare Facilities Master Plan 2013–2033 (General Secretariat, Supreme Council of Health)	General	Yes	GR, EQ	AL, F
Qatar National Health Strategy 2018–2022 (Ministry of Public Health)	General	Yes	EQ, HR	AL, AcW
Qatar National Vision 2030 (General Secretariat for Development Planning)	General	Yes	EQ, HR	AL
Rules & Regulations for Medical Examination of Expatriates Coming to GCC States for Residence (Gulf Health Council)	General	No		AM
Qatar Public Health Strategy 2017–2022 (Ministry of Public Health)	General	No	EQ	AL
Occupational safety and health policy in the State of Qatar (Ministry of Administrative Development, Labour and Social Affairs (MADLSA) and the Ministry of Public Health (MoPH))	General	No	EQ, HR	AM, AcW
Primary Health Care Corporation Corporate Strategic Plan 2019–2023: A Healthier Future for Our Families (Primary Health Care Corporation)	General	No	EQ	AL
Qatar Second National Development Strategy 2018 ∼ 2022 (Ministry of Development, Planning and Statistics, Qatar National Vision)	General	Yes	GR, EQ, HR	AL, AcS
Continuing Care Design Strategy (Ministry of Public Health)	General	No		AL
Law No. 19 of 2020 amending certain provisions of Law No. 21 of 2015 related to organizing the entry and exit of expatriates and their residence (Government of Qatar)	General	Yes	HR	AH
Law No. 18 of 2020 amending certain provisions of Labour Law No. 14 of 2004 (Government of Qatar)	General	No	HR	AH
Law No. (17) of 2020 Determining the National Minimum Wage for Workers and Domestic Workers (Government of Qatar)	General	No	HR	AH
Labour Inspection Policy—State of Qatar (Ministry of Administrative Development, Labour and Social Affairs (MADLSA))	General	Yes	HR	AM, AcW
Strategic Plan for Prevention and Control of Sexually Transmitted Infections, 2020–2024 (Draft)	STIs	Yes	GR, EQ, HR	AL
Operational plan for prevention and control of sexually transmitted infections 2020–2024 (Draft) (Ministry of Public Health)	STIs	Yes	EQ, HR	AL, AcW
COVID-19 Qatar National Response Action Plan (Ministry of Public Health)	COVID-19	Yes	EQ	AL
COVID-19 Vaccine (Ministry of Public Health)	COVID-19	Yes		AL
COVID-19 Preventative measures (Qatar Government Communications Office)	COVID-19	Yes	EQ	AL
Travel (Qatar Government Communications Office)	COVID-19	Yes		AL
Education (Qatar Government Communications Office)	COVID-19	No		AL
Vaccination Campaign (Qatar Government Communications Office)	COVID-19	No	EQ	AL
National Interim Guidelines Quarantine Measures for COVID-19 Containment (Ministry of Public Health)	COVID-19	No	EQ, HR	AL
Key Information for Workers (The Ministry of Administrative Development, Labour and Social Affairs)	COVID-19	Yes	EQ, HR	AL

a Policy authority level: AL = low policy authority (national action plans, plans, strategies, guidelines, standards, action plans, directives, activities, conventions, contracts, etc.); AM = medium policy authority (rules, regulations, policies, by-laws, conventions, etc.); AH = high policy authority (constitutions, acts, laws, decrees etc.).

Presence of accountability mechanism: AcW = weak accountability mechanism present (national agency specified and assigned responsibility for reporting in the public domain), AcS = strong accountability mechanism present (national agency specified and assigned responsibility for reporting in the public domain, mechanism for independent monitoring on progress is described and remedial actions if implementation does not occur are outlined).

Presence of financing mechanism: F = financing mechanism present (mention of named financing body or resources allocated, such as budget line item, for implementation of the policy).

Two Pakistani policies and 15 Qatari policies contained globally recommended interventions. Four global recommendations for migrant health were not addressed in either country’s policies (see Table 2). This included ensuring migrants’ access to grievance mechanisms for labour rights abuses; responding to gender inequities affecting migrant workers; enforcing national laws in line with international labour standards and adopting measures to target especially vulnerable migrant workers such as trafficked, undocumented and disabled migrants. Both countries failed to include most of the COVID-19-specific global recommendations relevant to labour migrants (Table 1).

Policies in both countries scored low on measures of health equity, human rights and gender, although Pakistan’s policies performed marginally better on these variables. One quarter of Pakistan policies and fewer than 1 in 10 Qatar policies refer to gender or the gendered demographics of the migrant population. Recognition of the intersection of equity and rights with migration was also limited. Fewer than a third of the Qatar policies referred to equity and a quarter took human rights into consideration, while no Pakistani policies referred to equity or rights.

**Figure 1.**
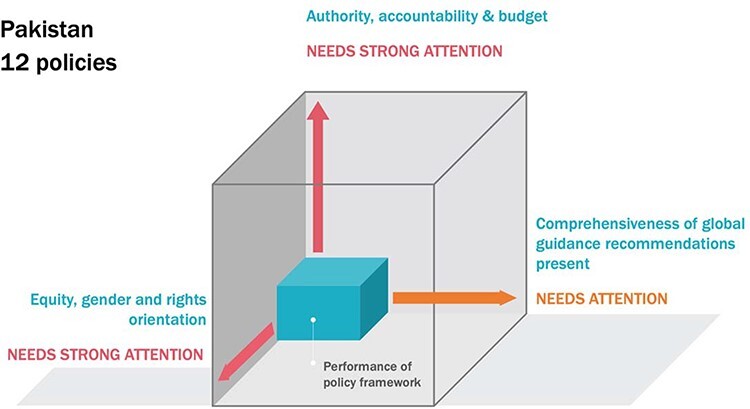
Analysis of national policies for the health of labour migrants: Pakistan

**Figure 2.**
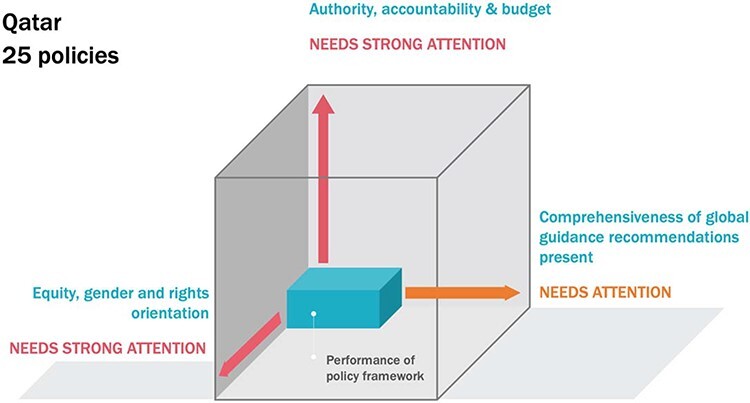
Analysis of national policies for the health of labour migrants: Qatar

The majority of policies (more than 8 in 10 in Pakistan and almost three-quarters in Qatar) were low-authority and also contained no reference to accountability mechanisms nor identified how to finance policy measures. Figures 1 and 2 represent the summary findings from each country within an illustrative ‘policy cube’. The three axes of each cube represent the strength of the policy framework to address the structural and social determinants of the health of labour migrants—comprehensiveness, values and equity considerations and implementation mechanisms. A more ‘full’ cube indicates a more robust policy framework in the country, and conversely, ‘deficit’ along any axis indicates an area where more attention is required.

### Stakeholder responses

We present stakeholder responses using the four categories identified by Shiffman and Smith (2007) as being associated with agenda-setting in policy formulation.

#### Ideas

An overarching framing of labour migrants in both countries was to regard them as a temporary population, first and foremost an expendable resource, rather than rights-holding members of society. In Pakistan, migrants were viewed as ‘people who bring remittances, cash stock, [to a] resource constrained economy’ [Pakistan, Civil Society, M]. While in Qatar, a civil society representative noted that there was an absence of ‘recognition of them beyond the work they do, beyond the transactional relationship’ [Qatar, civil society, W].

In Qatar, the same civil society respondent suggested that migrant workers were perceived by policymakers ‘as temporary’—i.e. operating with the idea that migration itself is a short-term issue, which resulted in an absence of ‘more permanent ways of dealing with migration.’

In both Pakistan and Qatar, labour migrant health responses were seen to be focused primarily on occupational health and injuries, with infectious diseases sidelined—partly due to stigma, especially STIs/HIV, leading to reservations around discussion: ‘Stigma … makes it very difficult for people to speak about HIV and about sex in general. We tend to deny that we have sex workers … men who have sex with men, [or] injecting drug users … Looking at it in this way puts younger generations [at] real risk [of HIV].’ [Qatar, government, M].

Where infectious diseases are addressed, it tends to be framed by Qatari officials through a security lens, with the priority being protecting the Qatari population from ‘imported’ diseases. As one respondent remarked: ‘[Infectious disease] screening is the equivalent of our wall’*—*referring to the wall US President Trump was then constructing on the Mexican border [Qatar, government, M].

#### Actor power

The framing of labour migrant health has played a role in determining which actors are engaged on the issue and which interventions should be prioritized. One respondent noted that curative medicine was ‘far better recognised and funded’ [Qatar, government, M] than preventive approaches. Interventions addressing social and structural determinants of health were seen as inappropriate due to a perception that ‘if you start talking about the social determinants of health, then you’re getting into other domains which the ministry of health … doesn’t have control over’. At the time of the interviews, the Ministry of Labour in Qatar was not active on migrant health beyond occupational health and safety.

Conversely, in Pakistan, it was noted that *‘*if you … look at the institutional arrangements of Pakistan’s system of migration, you do not find the Health Ministry there’ [Pakistan, Civil Society, M]. Stakeholders in Pakistan cited weak coordination between actors at national and subnational levels and its impact on the neglect of structural drivers of migrant health. Since devolution of power to the provincial level in 2010, there has been a lack of consensus about roles and responsibilities, including around migrant health: ‘The confusion persists about who is supposed to do what, whether the provinces are responsible for labour and manpower issues, whether the federal [or provincial] government should provide the laws’ [Pakistan, Multilateral, M].

Limited cooperation between the governments of Pakistan and Qatar on health was thought to have further mitigated against cohesive policy actions. For example, failures in information exchange were noted with respondents reporting that Qatar was not sharing medical reports on migrants’ infectious disease status with the Pakistan Government upon repatriation. Interviewees commented on the Pakistan embassy in Qatar’s ‘unwillingness to help’ [Qatar, civil society, W] to protect the rights of Pakistani migrants in the country, compared with embassies of other sending countries including Bangladesh and Nepal: ‘Once you arrive and your residency … is tied to the destination country, the buck stops there. The responsibility is with that country and with your employer, because under kafala that migration is outsourced to an individual’ [Qatar, civil society, W].

Stakeholders in both countries cited a lack of civil society mobilization and pressure as likely partially responsible for lack of government engagement on migrant health. It was remarked that in the GCC, ‘… there is no civil society activism, there aren’t any NGOs or legal aid. People often depend on their compatriots for help and support’ [Qatar, civil society, W]. In Pakistan, reliance of non-governmental actors on external institutions for funding was viewed as a barrier to civil society engagement on the issue: ‘The … available resources [for infectious disease] are only those … coming from the Global Fund’ [Pakistan, Civil Society, M].

In both countries, it was thought that the lack of government leadership on this issue was in part due to a reliance on the private sector and other non-state actors to take responsibility to protect the health of migrants. In Pakistan, discussion of health screening pre-departure was noted by one Government respondent as ‘we hold the recruitment agent responsible’ [Pakistan, Government, M], while at the start of the COVID-19 pandemic, it was noted by one public health respondent in Qatar that screening at the airport was in the hands of the private sector alone.

#### Issue characteristics

Limited availability of health data disaggregated by migration status was perceived as contributing to low prioritization: In Qatar, ‘disaggregated data is quite difficult to come by’ [Qatar, civil society, W] and respondents felt that migrant health data are often excluded from national health statistics, both factors that interviewees cited as obstacles to the issue being adequately appreciated to drive policymaker interest.

In both Pakistan and Qatar, stigma surrounding STIs/HIV was considered to be a key factor impeding accurate data collection and sharing: ‘[The Qatari government] completely deny HIV because of the string of stigma and religious elements attached. If you ask them how many HIV cases there are, they would say zero. We have no information’ [Pakistan, Multilateral, M].

It was reported that migrants’ fear that testing positive for an infectious disease would jeopardize their employment or visa—thus contributing to a potential under-reporting of the ‘true’ prevalence of HIV/STIs. ‘[Migrant workers] are worried, they don’t want to admit they are [sexually active] … They don’t want to lose their job’ [Qatar, government, M]. One respondent also highlighted limited data on infections among undocumented migrants on account of their exclusion from national health systems, ‘If you have a health card …, highly subsidized health care, [and] a Qatar ID and then you’re using the services, but if you do not have proof of your … legal residence, then [health-care workers’] have to inform the government’ [Qatar, civil society, W].

One of the global recommendations involved ‘Cooperation between governments … on managed labour migration and health programmes’ (Table 2). Mechanisms for enhancing such cooperation could include bilateral agreements—these can act as a tool for countries to hold one another accountable for protecting the rights of migrants by placing (legally binding) conditions on the sending and receiving of migrants for employment purposes. However, the issue characteristics associated with such agreements were perceived by some informants to limit their feasible or effectiveness. As one respondent pointed out, ‘Pakistan [is] … totally dependent on these workers’ [Pakistan, Civil Society, M]—leaving the country in a weaker negotiating position vis-a-vis bilateral agreements. Moreover, some respondents felt that when such agreements have been implemented in the region, they have sometimes had negative impacts on labour rights: ‘Bilateral agreements … tend to be … a race to the bottom because each country is trying to make itself a more attractive source country for labour. So they will … reduce their expectations in the hope that they can increase the number of migrants that are being sent’ [Qatar, civil society, W].

#### Political contexts

Pakistan was perceived to be in a weak position to negotiate better protections for migrants’ health and rights from host countries, a situation exacerbated by the impact of the COVID-19 pandemic, which resulted in the repatriation of a large number of migrants to Pakistan. ‘[T]he State of Pakistan is desperate [for] money to fix its balance of payments … the macroeconomic indicators are the only concern—but the Pakistan Government must negotiate with the governments of the Middle East to enforce certain protection[s] and some better working conditions’ [Pakistan, civil society, M].

Respondents suggested that greater regional coordination between countries on infectious disease would be important to improve interventions on migrant health: ‘Never, ever, [have] we met together, for example, to discuss what we would do in tuberculosis [among] migrants. As a GCC, no. Every country has his own policy’ [Qatar, government, M]. Similarly, it was thought that ‘three or four countries in South Asia [could] collectively raise their voice in a more systematic, strengthened way. Regional block formation from the sending countries [is] a first step [to improve the] health and living conditions of the labour migrants’ [Pakistan, multilateral, M].

## Discussion

Labour migrants are integral to the economy of many countries. The estimated 169 million international migrant workers globally leave their home countries driven by a range of factors, including lack of labour supply in destination countries and a dearth of economic opportunities in sending countries—referring to the so-called ‘reserve army of labour’ in Marx’s critiques of the capitalist organization of work—where remittances can constitute an important component of the economy (ILO, 2021a). The climate emergency (IOM, 2021) and increasing economic inequalities (United Nations, n.d.) are also contributing to more people on the move for work (IOM, 2021).

It is not just national economies that rely on the movement of human labour. In a [men’s] football World Cup year (2022), where the governing body has an estimated annual revenue of over USD four and a half billion (FIFA, 2020), it is inconceivable that the tournament would be going ahead without migrants who have built the tournament’s infrastructure in Qatar. But as has been pointed out, the World Cup is a ‘multinational project’ rather than just a Qatari one (Babar and Vora, 2022), and as such, addressing the rights of labour migrants to health and well-being requires attention to policies and actors both within Qatar and beyond its borders.

Ensuring that the health and well-being of international labour migrants is protected within a ‘migration ecosystem’ requires action on social and structural determinants of health. Our review found a large amount of global policy guidance that could be enacted to protect the health and well-being of labour migrants through action on structural/social determinants. However, our policy analysis in Pakistan and Qatar revealed considerable room for improvement within this policy ecosystem. Qatar in particular had a substantial number of policies containing commitments to structural interventions. However, the policy cube method of policy analysis (Buse *et al.*, 2020) allowed us to go beyond policy content and examine issues of equity and accountability, among other policy factors. In both Pakistan and Qatar, policies tend to have low potential for being implemented and do little to address human rights, health equity and equality for migrant workers.

Interviews with stakeholders in both countries has revealed a range of reasons why policies may be lacking or poorly implemented—including questions of who is responsible for the well-being of labour migrants and who has the power to affect positive interventions. Whilst interviewees attributed the limited attention to labour migrants with/at risk of STIs/HIV to ‘cultural conservatism’, a similar lack of attention found in COVID-19 policies in both Pakistan and Qatar suggests that it is not entirely explained by sex- or disease-specific stigma but may instead reflect a neglect of the health of migrant populations more generally.

What explains these deficits in policy attention being paid to the health of labour migrants, including through addressing the structural determinants of health? One factor that may contribute is the framing (‘ideas’ in the Shiffman/Smith framework) of this population as, principally, a source of economic resources rather than individuals with rights to be respected, protected and fulfilled. Framing was also considered to be behind the relative lack of consideration of health as dependent on structural drivers, not just a question of access to health-care services—an issue that is not limited to the subject of this research. More specifically, although the bulk of health is created outside of the health-care sector in all countries, health is typically framed primarily as a responsibility of ministries of health rather than as a responsibility of systems governing social protection, employment law, housing, nutrition, education or transport, etc., which ultimately determines most health outcomes (Buse, 2021; 2022). As such, attention to the health of migrants becomes conflated as the preserve of ministries of health alone as opposed to their work in collaboration with other ministries.

Second, power inequalities appear to be contributing to policy inaction. One UN Special Rapporteur in 2020 described Qatar’s laws, policies and practices as constituting system of ‘structural racial discrimination against non-nationals in Qatar’ in which ‘national origin and nationality determines the extent of their enjoyment of their human rights’ (UNHRC, 2020). In Pakistan, one interviewee in our study voiced an opinion that policy was made by ‘elites [in Pakistan] in cahoots with the elites in this country [Qatar]’—leading to a neglect of migrant health and rights since ‘you know, it is not a concern for them, right’, reflecting both power imbalances between nationals and non-nationals and imbalances between the ruling classes and the working class. Other interviewees identified unequal power dynamics between sending and host countries as contributing to the reluctance of Pakistani Government officials in raising issues of labour migrants’ health rights in bilateral negotiations.

Third, a lack of engagement of key actors on the issue of migrant health emerged as another reason for the deficiencies in policy content. Civil society groups and the media were noted to not be playing a powerful role in this space (beyond highlighting some individual cases of migrants stranded during the COVID-19 pandemic). Moreover, the absence of key government ministries and weak cooperation between health and non-health actors on migration included, e.g. Pakistan’s Ministry of Health missing from ‘institutional arrangements of Pakistan’s system of migration’ [Pakistan, government, M]. Instead, it was noted by several respondents that one set of powerful actors in the system was the private agencies that labour migrants often pay substantial fees to. These private sector actors include employment promoters, who recruit workers for jobs advertised by foreign employers (ILO, 2016b), and others who exact costs for visa applications, international travel and medical tests on to the migrants themselves—meaning they have often accumulated substantial debts by the time they arrive in the destination country (Jureidini, 2017a). Despite our best efforts to interview these core stakeholders, we were unable to secure any interviews with them.

Fourth, there appears to be a gap in the availability of (disaggregated) migrant health data (i.e. a deficit in the so-called ‘issue characteristics’). Interviewees in Pakistan noted that health surveillance systems were generally weak, and for both COVID-19 and HIV/STIs, data disaggregated by migration status were not perceived as a priority for the surveillance systems. Interviewees in Qatar voiced concern that the absence of fully disaggregated data on migrant health meant the issue could be overlooked in policy formulation. This absence of data has been raised as a concern previously—Qatar’s Planning and Statistics Authority has been criticized for not presenting data in a form that enables analysis across population groups (Amnesty International, 2021). A ‘lack of disaggregated statistical data on the ethnic and racial composition of the population, both among Qataris and non-Qataris’ was found to be an obstacle to understanding the extent of racial discrimination in the country by the UN Special Rapporteur on contemporary forms of racism (UNHRC, 2020), and Pakistan’s Bureau of Emigration and Overseas Employment (BEOE), the main monitoring body for overseas migration, has been noted to not disaggregate data by gender (ILO, 2020).

Finally, the study has highlighted what appears to be a lack of implemented inter-country action to address key structural determinants of the health and well-being of labour migrants moving from one country to another. Pakistan and Qatar have bilateral agreements on labour migration that have been in place for decades; for example, an ‘Agreement and Additional Protocol regulating the employment of Pakistani workers in the State of Qatar’ (Government of Pakistan and Government of Qatar, 1992) came into force in 1992, and its articles cover several areas relating to workers’ rights (e.g. access to employment information), as well as the establishment of a ‘Joint Committee … [to] coordinate between the Governments of Qatar and Pakistan’. However, despite such bilateral memorandums of understanding and agreements being in place, none of the stakeholders (in Pakistan or Qatar) recognized these as a source of protection for workers’ health and well-being or a functioning system of coordination and accountability.

### Policy reform is possible

As we have seen throughout this paper, policy reform has been underway in Qatar for some time. International criticism of Qatar’s treatment of labour migrants, combined with the political will for reform, appear to have played an important role in driving change. Following the 2017 signing of a technical cooperation accord with ILO, in which the government agreed to set a minimum wage, the International Covenant on Civil and Political Rights and the International Covenant on Economic, Social and Cultural Rights were ratified in 2018 and changes to the *kafala* system of sponsorship made in 2020 (Abraham, 2021). The revoking of the *kafala* system has itself operated as a policy window by providing an opportunity to formalize new legal measures to protect the labour rights of migrant workers.

Likewise, in Pakistan, steps have been taken in recent years towards reforming labour migration policy. In 2017, Pakistan’s BEOE signed an agreement with State Life Insurance Corporation of Pakistan to extend the insurance coverage duration for migrants from 2 years to 5 years (BEOE, 2017). In 2017, the BEOE also signed an Memorandum of Understanding with the National Database & Registration Authority on the introduced digital registration and biometric verification for all migrants, in an effort to ensure transparency and streamline the registration process (Bureau of Emigration & Overseas Employment, Pakistan, 2017). They also set out to develop country-specific flyers and videos for departing migrants to increase access to free, comprehensive and accurate information for migrants regarding their rights and their recruitment and employment conditions (Bureau of Emigration & Overseas Employment, Pakistan, 2017).

One avenue towards strengthened government commitments to protect the health and rights of labour migrants may be to build the influence of non-governmental actors, including trade unions and civil society groups. These approaches have been effectively utilized to push for the realization of health and welfare rights for migrants by trade union bodies in both origin countries, including Indonesia, Nepal and Thailand, and destination countries, including Malaysia (ILO, 2014). In Nepal, as a result of years of lobbying by GEFONT (General Federation of Nepalese Trade Unions), the government adopted the New Labour Act in 2017, which extended labour protections and social security systems to all workers, including migrant workers, established a minimum wage and legislated against pay discrimination (ILO, 2014; 2019; IDWF, 2017).

Another approach that has been used to strengthen government measures to protect migrant workers health and welfare utilizes formal bilateral agreements. Although (as noted) these exist between Qatar and Pakistan, their apparent lack of implementation hampers their utility in ensuring that migration takes place in accordance with global norms and standards (ILO, n.d.a). In contrast, The Philippines, which has 12 bilateral agreements with labour-receiving countries and one with a labour-sending country (ILO, n.d.b), has used these agreements to seek to protect Filipinos working overseas by making them one of four conditions that a host country must meet in order to receive Filipino workers (Wickramasekara, 2015)—thus providing a working model for consideration.

### Policy reform is not enough

Our analysis has shown several commitments made by national authorities (particularly Qatar) to address the determinants of labour migrant health, but the relative absence of considerations of equity or implementation potential in the policies raises concerns about the delivery in practice—a feature our study was not designed to address. This highlights the need to develop systems of independent monitoring and accountability alongside progressive policy reform.

## Limitations

There are a number of limitations to the study, not least of which is that we were unable to interview labour migrants themselves in either Pakistan or Qatar. We were, however, able to undertake a substantive programme of public engagement in Pakistan with labour migrants who had worked (or were working) in a number of GCC countries, and their experiences and stories [reflected through collaboration with artists and presented in an online gallery (Selma Project, 2020)] align closely with many of the interviewees’ comments, particularly in Pakistan.

A second limitation was due to the impact of the COVID-19 pandemic shortly after we completed our first round of interviews in Qatar. The pandemic meant that we were unable to complete a planned second round of interviews in the country, which means that the focus of the interviews was predominantly on HIV/STIs, while those in Pakistan (conducted from July 2020 onwards) included both HIV/STIs and COVID-19 as topics of interview focus.

Finally, this study has focused on one sending and one destination country; the findings do not necessarily generalize to other settings. However, we expect that the broad findings—namely a double jeopardy comprising the neglect by governments of labour migrant health and of structural-level interventions to improve labour migrant health outcomes—likely apply to other countries that send and host migrant workers. Similarly, the scope of this paper is limited to migrant labourers, so its findings do not necessarily apply to policies targeting other labour migrants such as white-collar workers or domestic workers (who are predominantly female migrant workers).

## Conclusions

Despite a relatively large number of global recommendations, our review of national policies relevant to labour migrant health in a country of origin and destination found notable gaps to address upstream determinants. Approximately one third of interventions recommended in global guidance are not addressed in policies of either Pakistan or Qatar, and policies present in health and non-health sectors for COVID-19 and HIV/STI policies tended to have weak implementation potential and little consideration of rights or equity.

Driving progress on the health equity, welfare and rights of migrant workers, a population group that will continue to grow, will depend on the mobilization of actors across the public health system—including governments, civil society, multilaterals and academics—on the social and structural determinants of health. Governments of both host and sending countries should be prioritizing structural determinants in policies and engaging key domestic actors, including from civil society, to drive a coordinated, evidence-informed response. Multilateral actors must continue to push for more widespread understanding of the existing raft of recommendations for the health of labour migrants and establish systems for independent monitoring and accountability. Civil society actors need to be engaged in the development of this response and resourced to enable them to hold governments accountable for its implementation. For the academic sphere, more research is needed to build the evidence base on the effectiveness of structural interventions, which are currently almost entirely overlooked in favour of interventions (and research) targeting the individual level.

Realizing environments that are conducive to the health and well-being of labour migrants demands designing policies that align better with the evidence base, as well as a shift in policymakers’ perceptions and framings of migrants that sees them as people deserving of equality, rights and respect. The disregard of labour migrants’ rights, evident in national policies, in decision maker attitudes and the outsourcing responsibility to the private sector, makes a mockery of commitments made by UN Member States to leave no one behind (UNSDG, n.d.). As UN Secretary-General António Guterres posited in the negotiations leading to the adoption of The Global Compact for Migration, ‘Today, one of the single most fundamental determinants of the capacity of individuals to realize their full potential and rights is their place of birth … Migration, properly managed, is a route for individuals to … achieve the dignity that our predecessors enshrined in the Universal Declaration of Human Rights. Their quest for equality is a legitimate one’ (United Nations General Assembly, 2017).

## Data Availability

The data underlying this article cannot be shared publicly due to questions of privacy for the participating interviewees. Their participation in the study was on the basis of confidentiality and anonymous reporting.

## Endnotes

1. Included draft policy documents shared by key informants not yet published.

2. International treaties signed (and ratified) by the country were not included.

3. This included documents produced during the COVID-19 pandemic or pre-dates COVID-19 but refers to pandemic control.

4. In Pakistan, health policy is devolved to the provincial level. However, no significant differences in policy commitments to migrant health were noted between provincial and federal level policies. Therefore, only federal level policies were included in the sample.

## Reflexivity Statement

Our multi-country, multidisciplinary team of authors comprises a mix of genders (four women and one man), geographies (Pakistan, UK), levels of seniority, ages and other intersectional aspects of identity. We have expertise in policy analysis, gender analysis, international human rights, public health and public engagement. Four of the five authors have conducted policy research in Pakistan, and two of the authors conducted policy interviews in Qatar. Three of the five team members were able to undertake field work in Qatar before COVID travel restrictions came into force.
